# Therapeutic Potential of Naturally Occurring Small Molecules to Target the Wnt/β-Catenin Signaling Pathway in Colorectal Cancer

**DOI:** 10.3390/cancers14020403

**Published:** 2022-01-14

**Authors:** Luiz F. S. Oliveira, Danilo Predes, Helena L. Borges, Jose G. Abreu

**Affiliations:** Programa de Biologia Celular e do Desenvolvimento, Instituto de Ciências Biomédicas, Universidade Federal do Rio de Janeiro, Rio de Janeiro 21941-902, Brazil; danilopredes@gmail.com (D.P.); hborges@icb.ufrj.br (H.L.B.)

**Keywords:** small molecules, anticancer drugs, target therapy, natural compounds

## Abstract

**Simple Summary:**

Colorectal cancer (CRC) is an emerging public health problem and the second leading cause of death worldwide, with a significant socioeconomic impact in several countries. The 5-year survival rate is only 12% due to the lack of early diagnosis and resistance to available treatments, and the canonical Wnt signaling pathway is involved in this process. This review underlines the importance of understanding the fundamental roles of this pathway in physiological and pathological contexts and analyzes the use of naturally occurring small molecules that inhibits the Wnt/β-catenin pathway in experimental models of CRC. We also discuss the progress and challenges of moving these small molecules off the laboratory bench into the clinical platform.

**Abstract:**

Colorectal cancer (CRC) ranks second in the number of cancer deaths worldwide, mainly due to late diagnoses, which restrict treatment in the potentially curable stages and decrease patient survival. The treatment of CRC involves surgery to remove the tumor tissue, in addition to radiotherapy and systemic chemotherapy sessions. However, almost half of patients are resistant to these treatments, especially in metastatic cases, where the 5-year survival rate is only 12%. This factor may be related to the intratumoral heterogeneity, tumor microenvironment (TME), and the presence of cancer stem cells (CSCs), which is impossible to resolve with the standard approaches currently available in clinical practice. CSCs are APC-deficient, and the search for alternative therapeutic agents such as small molecules from natural sources is a promising strategy, as these substances have several antitumor properties. Many of those interfere with the regulation of signaling pathways at the central core of CRC development, such as the Wnt/β-catenin, which plays a crucial role in the cell proliferation and stemness in the tumor. This review will discuss the use of naturally occurring small molecules inhibiting the Wnt/β-catenin pathway in experimental CRC models over the past decade, highlighting the molecular targets in the Wnt/β-catenin pathway and the mechanisms through which these molecules perform their antitumor activities.

## 1. Introduction

According to the World Health Organization (WHO) definition, cancer is a general term for a group of diseases characterized by changes in cell proliferation that can affect any part of the body, invade other tissues, and cause death [1]. More than 19 million new cancer cases are diagnosed each year, making it the second leading cause of death globally, with approximately 10 million deaths each year [2]. The most common cancer subtypes are breast cancer (11.7%), lung cancer (11.4%), and colon cancer (10%), with more than 2 million new cases diagnosed in 2020 [2,3].

Colorectal cancer (CRC) has a high incidence and mortality rate and is the second leading cause of cancer deaths worldwide [3,4]. Environmental and hereditary risks play a crucial role in the development of the disease [4,5]. In this case, lifestyle includes an important group of changeable risk factors, particularly weight gain, sedentary lifestyle, smoking, excessive drinking, unhealthy diet, and excessive consumption of red meat [6,7,8]. In addition, patients with a history of inflammatory bowel disease or polyps are more likely to develop this disease [5,9].

Early diagnosis allows family follow-up and the adoption of appropriate medical conduct for better chances of cure [10]. However, access to screenings is insufficient in most countries, which impairs early detection [11,12,13]. Stages I and II CRC are potentially curable when diagnosed and treated early, with 5-year survival between 70% and 90%. Nevertheless, this rate decreases in stage III and stage IV, especially in metastatic cases, which have only 12% 5-year survival [10,14,15,16]. Poor prognosis in advanced/metastatic CRC cases is mainly linked to its high heterogeneity in addition to the presence of cancer stem cells (CSCs) [16,17]. CSCs are capable of self-renewal and symmetrically divide into two CSCs or one CSC and one daughter cell, which increases excessively the cell growth and supports tumor development [16,17].

CSCs share some regulatory mechanisms of normal intestinal stem cells (ISC), which generate one of the main therapeutic challenges because CSC-targeted therapies may inadvertently harm ISC homeostasis or stem cells in bystander organs [17,18]. The Wnt/β-catenin signaling pathway is a prime regulator of stemness in intestinal crypts [19,20,21,22]. Interestingly, several studies have shown that this pathway is closely associated with chemoresistance in CRC patients [23,24,25,26]. Thus, the comprehension of molecular mechanisms that regulate tumor maintenance is a golden chance to improve current treatments and increase overall survival and reduce tumor recurrence in CRC patients [17]. In this review, we discuss the latest research using naturally occurring small molecules-based inhibition of Wnt/β-catenin signaling pathway components in CRC models, as well as the advances and challenges to make the Wnt/β-catenin signaling pathway a druggable target for CRC.

## 2. The Wnt/β-Catenin Signaling Pathway

The cell signaling by the Wnt family of secreted glycoproteins is one of the fundamental mechanisms that regulate cell proliferation, polarity, and cell fate [27,28,29]. The signaling promoted by the 19 Wnt glycoproteins can be subdivided into the Wnt/β-catenin pathway, also known as the Wnt canonical pathway, and the non-canonical Wnt pathway, which does not depend on β-catenin, that encompasses the Wnt/planar cell polarity (Wnt/PCP), Wnt/Ca2+ pathway, and the Wnt-dependent stabilization of proteins pathway (Wnt/STOP) [30].

**Figure 1:**
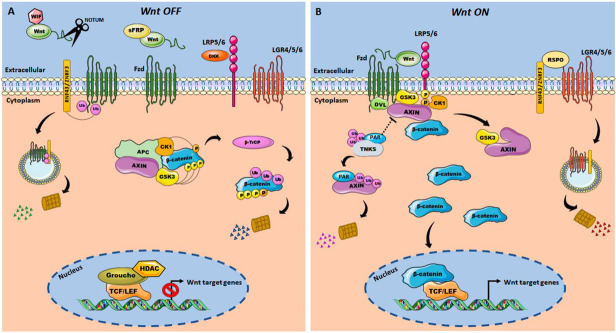
Schematic representation of the regulatory model of the Wnt/β-catenin signaling pathway. (**A**) in the absence of Wnt; (**B**) after binding of Wnt ligand. WIF, Wnt Inhibitor Factor; sFRP, Secreted Frizzled-related Protein; DKK, Dickkopf; LRP, LDL Receptor-related Protein; APC, Adenomatous Polyposis Coli; CK1, Casein Kinase 1; GSK3, Glycogen Synthase Kinase-3; Dvl, Disheveled; Tnks, Tankyrases; β-TrCP, Beta-Transducin repeat-Containing Protein; β-cat, β-catenin; TCF, T-Cell Factor; LEF, Lymphoid Enhancer-binding Factor; HDAC, Histone Deacetylase; TLE, Transducin-Like Enhancer Proteins; PAR, Poly-(ADP-ribose); RSPO, R-spondin; ZNRF3, E3 ubiquitin-protein ligases; RNF43, Ring Finger Protein 43; LGR4/5/6, Leucine-rich repeat-containing G-protein coupled Receptors 4/5/6; P, phosphorylation; Ub, ubiquitination.

The canonical Wnt signaling pathway relies on the fine balance of β-catenin protein level [29,31]. In the absence of Wnt ligands, the ubiquitin-dependent proteasomal degradation keeps the β-catenin protein at basal levels [32]. This process is promoted by a protein complex known as the β-catenin destruction complex, in which the Axin protein acts as a scaffold for the tumor suppressor Adenomatous polyposis coli (APC), Casein kinase 1 (CK1), and Glycogen synthase kinase-3 α/β (GSK-3α/β) [33,34,35,36,37]. Thus, Axin interacts with β-catenin bringing it closer to CK1, which phosphorylates it at serine 45, whereas GSK-3β phosphorylates threonine 41 and serine 37 and 33 [38,39], addressing it for ubiquitination by the protein β-Transducin repeat-containing protein E3-ubiquitin ligase (β-TrCP), and further degradation via the proteasome [40]. This process allows the Groucho protein to bind to T-Cell specific transcription Factor/Lymphoid Enhancer-binding Factor (TCF/LEF), thus blocking the transcriptional promoters and enhancers of the canonical Wnt pathway. Thus, the TCF/Groucho complex suppresses the transcription of target genes of the canonical Wnt signaling pathway [41,42] (Figure 1A).

Wnt proteins are crucial in the canonical signaling pathway as well as in polarized epithelial cells [43]. Excluding WntD (dorsal Wnt inhibitor in *Drosophila*), all Wnt proteins are palmitoylated in the endoplasmic reticulum (ER) [44,45,46] by ER-membrane-bound-O-acyl transferase Porcupine [47], leading to its recognition by Wntless (WLS) that transports the Wnt protein to the cell membrane, where it will be finally secreted [48,49]. Once released, Wnt ligands travel into the extracellular space to activate their signal transduction [45,46]. The binding of the Wnt proteins to the Frizzled-LRP5/6 complex in the membrane leads to the recruitment of the Disheveled protein (Dvl) through direct interaction with the Frizzled receptor [50,51]. This process culminates in the recruitment of the Axin-GSK3 complex to phosphorylate the PPPSPxS motifs of the intracellular domain of LRP5/6, preparing them for further phosphorylation by CK1γ [38,52,53,54]. Phosphorylation of LRP5/6 PPPSPxS motifs inhibits GSK-3β allowing the dephosphorylation of Axin by PP1 phosphatase, thereby assuming a closed conformation, incapable of interacting with β-catenin [55]. However, Tankyrases (TNKs) regulate Axin homeostasis through PARsylation and subsequent degradation by the proteasome [31,32,56]. Thus, β-catenin accumulates in the cytoplasm and translocate to the nucleus, interacting with the TCF/LEF and acting as a coactivator of the transcription of Wnt signaling target genes [57], which comprises cell cycle progression, stemness, and positive and negative feedback genes (Figure 1B).

Additionally, negative feedback mediated by the E3 ubiquitin-protein ligases ZNRF3 and Ring Finger Protein 43 (RNF43) promote the regulation of β-catenin through the degradation of LRP5/6 and Frizzled receptors, reducing the availability of binding sites for the Wnt protein, resulting in subsequent downstream β-catenin degradation [58,59]. However, the R-spondin (RSPO) proteins induce the indirect association between ZNRF3 and Leucine-rich repeat-containing G-protein coupled receptors (LGR) 4/5/6, promoting membrane clearance of ZNRF3 and RNF43, thereby enhancing the availability of the Wnt receptor in the cell membrane, which potentiates Wnt ligand-mediated activation of the Wnt/β-catenin pathway [58,59] (Figure 1B).

### Wnt/β-Catenin Pathway in Colorectal Cancer

The intestine is one of the most dynamic organs in the human body [18,19]. The strong renewal ability of intestinal epithelium allows the replacement of all the cell lining over its more than 7 m length every week, most likely in response to chemical, microbiological, and mechanical stresses caused by digestion and evacuation [19]. This replacement initiates at the bottom of the intestinal crypts, by asymmetric division, in a niche containing ISCs [18]. Several embryonic-conserved signaling pathways are involved in self-renewal, proliferation, and differentiation of the ISC niche, with the Wnt/β-catenin pathway acting as a prime regulator for the maintenance of the stem cell pool [19,20].

As the Wnt pathway is crucial for promoting homeostasis in the ISC niche, it is not surprising that changes in its components trigger pathological events [19,20,60]. Although the pathophysiological mechanisms that drive colorectal carcinogenesis are heterogeneous, the tumoral transformation of normal intestinal epithelial cells (IEC) depends on an event that triggers chromosomal instability (CIN) and the accumulation of somatic mutations and epigenetic alterations [20,61,62]. In this context, the deregulation of the Wnt/β-catenin signaling components is the most frequent event and leads to hyperproliferation in the intestinal crypt [63,64].

According to The Cancer Genome Atlas (TCGA), changes in Wnt/β-catenin signaling members such as APC are frequently the classic driver event in more than 90% of CRC cases [65]. The APC gene encodes a 310 kDa multidomain protein that is one of the prime regulators of β-catenin levels. Its exon 15 corresponds to approximately 75% of the APC coding sequence and is also a common region for its mutations [66,67]. Germ-line mutations in the APC gene are associated with familial adenomatous polyposis (FAP) and hereditary predisposition to develop CRC [68,69]. In addition, somatic mutations are found in more than 80% of sporadic CRC, and loss of heterozygosity (LOH) of chromosome 5q is reported in 30% to 40% of patients with CRC [70,71,72]. In both cases, the loss of APC promotes constitutive activation of the Wnt/β-catenin signaling pathway and the clonal expansion of the “initiated” cells leading to the appearance of a benign polyp with aberrant crypts [63,66,73].

**Figure 2:**
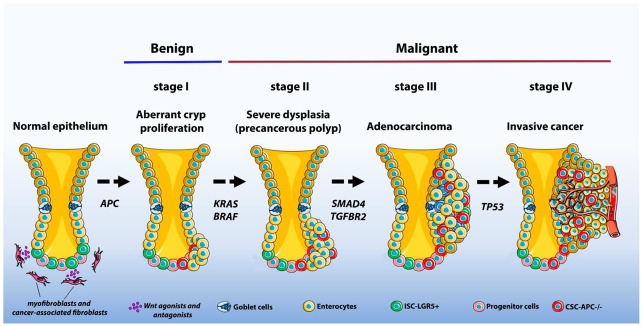
Schematic representation of the multistep process of carcinogenesis in the colonic epithelium with the corresponding genetic alteration in each step. The loss of APC leads to the development of tubular adenomas, which can progress to adenocarcinomas. APC, adenomatous polyposis coli; KRAS, Kirsten rat sarcoma viral oncogene homolog; BRAF, B-Raf Proto-Oncogene, Serine/Threonine Kinase; SMAD4, Mothers against decapentaplegic homolog 4; TGFBR2, Transforming Growth Factor Beta Receptor 2; TP53, tumor protein p53.

The increase of genomic instability and the successive mutations in genes such as KRAS and SMADs trigger the formation of dysplastic tubular adenomas [61,74,75]. As a result, alterations in the Transforming growth factor-β (TGF-β) pathway and the inactivation of the TP53 gene culminate in the formation of invasive adenocarcinomas [61,75,76,77]. Once epithelial tumor tissue is in constant interaction with cells in tumor microenvironment (TME) through the action of cytokines, chemokines, and growth factors, additional mutations, and changes in TME components give advantage to tumor clones and allow the tumors to later metastasize to distant organs [78,79,80,81,82] (Figure 2).

The constitutive activation of Wnt signaling triggered by the loss of APC confers mutant ISCs a clonal advantage, thereby enhancing the critical role of the Wnt signaling pathway in the development and progression of CRC [79,80,81]. In addition, APC-deficient ISC clones further increase their fitness through the modulation of the tumor microenvironment by secreting Wnt antagonists such as Notum. Secreted Notum inhibits the Wnt ligands necessary for the stemness of the neighboring wild-type ISC, therefore inducing their differentiation, which favors intra- and inter-crypt competition between APC-truncated and normal clones, conferring the cell growth advantage to CRC cells [79,80,81]. Intriguingly, colon cancer cells recover their normal function when wild-type APC levels are restored, triggering the differentiation, and restoring crypt homeostasis, even with severe oncogenic insults such as KRAS and p53 mutations [76].

These discoveries reinforce the importance of understanding the molecular mechanisms that implicate the Wnt pathway in the promotion and progression of CRC by the scientific community and draw attention to its potential for targeted therapies.

## 3. Systemic CRC Therapy and Wnt/β-Catenin Signaling Pathway

Treatment of CRC depends on the staging and may include laparoscopic removal of polyps or surgical removal of damaged areas of the intestine [83,84,85,86]. 5-Fluorouracil (5-FU)-based monotherapy is generally the first-line systemic approach used to treat frail or elderly patients [87,88,89]. On the other hand, in reasonably healthy patients, first-line therapy consists of combined regimens of 5-FU and oxaliplatin (FOLFOX or CAPOX) or regimens based on irinotecan (FOLFIRI or CAPIRI). In advanced and metastatic CRC, the treatment may include the FOLFOXIRI regimen, which is composed of 5-FU, leucovorin, oxaliplatin, and irinotecan simultaneously combined. However, studies have shown that this regimen is rarely used due to its potential for increased toxicity.

Although combination drugs for cancer treatment have improved overall survival compared to monotherapy, CRC recurrence remains the reality for approximately 40% of the patients after primary treated tumors [89]. The stemness in tumor cells is an important underlying mechanism that contributes to standard treatment resistance and tumor recurrence [17,90]. CSCs are a small population of undifferentiated tumorigenic cells within tumors that are capable of self-renewal and produce different cell clones responsible for the initiation and sustaining of tumor growth [90]. CSCs are APC-deficient [79,81]. The loss of APC leads to constitutive activation of the Wnt/β-catenin signaling pathway that sustains CSC survival and promotes the clonal exclusion of WT-ISC through the secretion of Wnt antagonists in the tumor microenvironment (TME) [79,80,81]. Because APC and other Wnt signaling members are critical to the development, progression, and recurrence of CRC, components of the Wnt/β-catenin signaling pathway may correspond to a promising source therapeutic targets for CRC treatment. In this review, we discuss the Wnt/β-catenin signaling components as a target for CRC therapies using naturally occurring small molecules, highlighting the mechanisms by which these molecules exert their antitumor effects.

## 4. Wnt/β-Catenin Components as a Target for Natural-Derived Small Molecules-Based Therapy in CRC

**Figure 3:**
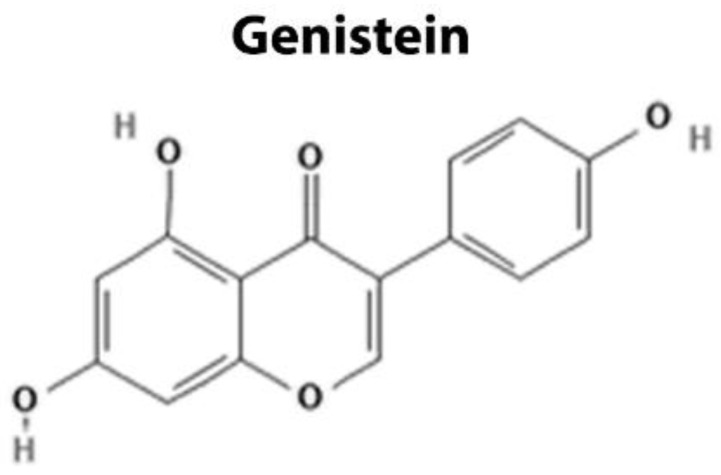
Chemical structure of Genistein, which is a naturally occurring small molecule that targets extracellular components such as Wnt antagonists in the Wnt/β-catenin signaling pathway.

**Figure 4:**
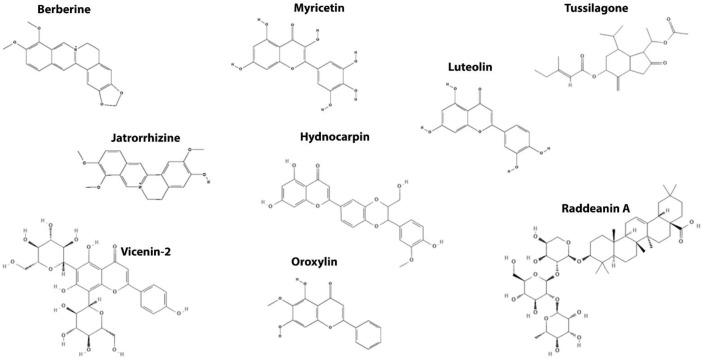
Chemical structures from the naturally occurring small molecules that target the destruction complex components in the Wnt/β-catenin signaling pathway.

**Figure 5:**
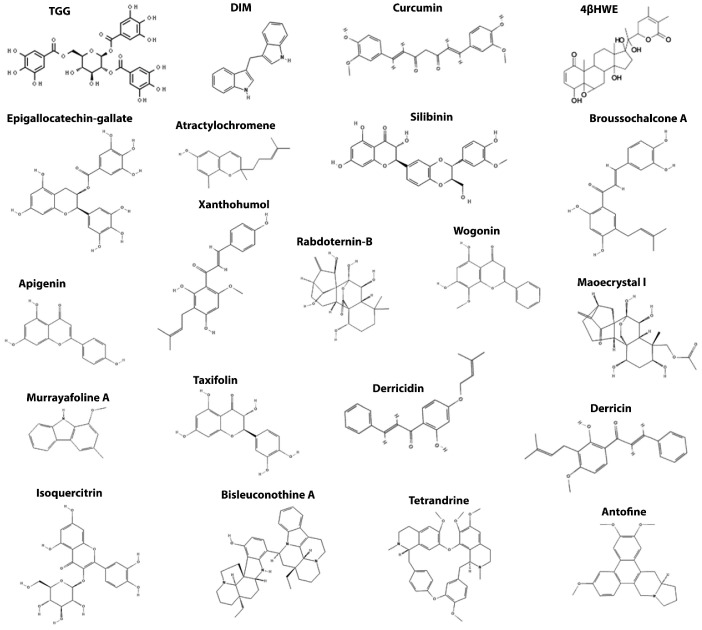
Chemical structures from the naturally occurring small molecules that target the β-catenin protein in the Wnt/β-catenin signaling pathway.

**Figure 6:**
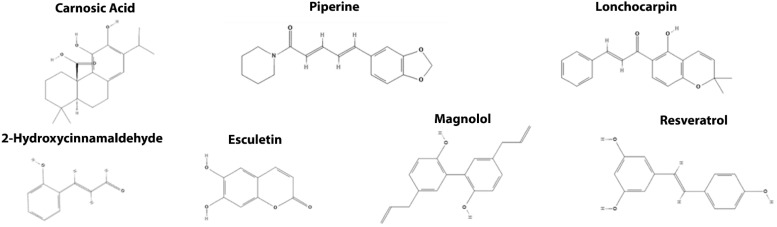
Chemical structures from the naturally occurring small molecules that target the β-catenin transcriptional activity in the Wnt/β-catenin signaling pathway.

Several FDA-approved anticancer drugs are based on natural sources such as natural compounds. The alkaloids vincristine and vinblastine are used as first-line therapy for leukemia and lymphomas [91,92], while paclitaxel is the first-line therapy for breast cancer [93]. Irinotecan, a second-line treatment for CRC, is a drug developed based on the alkaloid camptothecin [94]. Polyphenols are derived from natural sources and are widely used therapeutic alternatives for cancer treatment. These small molecules are synthesized by plants and are found in multiple sources such as seeds, leaves, and roots [95,96,97,98]. These molecules have many biological effects with significant therapeutic impact, including chemoprotective and antitumor properties related to their ability to interact with multiple signaling pathways components such as the Wnt/β-catenin pathway [96,99,100]. In this review, we highlighted dozen naturally occurring small molecules targeting the Wnt/β-catenin signaling pathway, such as extracellular proteins (Figure 3), components of the destruction complex (Figure 4), the β-catenin protein (Figure 5) as well as its transcriptional activity (Figure 6). In the following sessions, we present the molecular target, mechanisms of action (Table 1), and the anticancer effects of each molecule on different CRC models.

### 4.1. Targeting Extracellular Components

Genistein is one of the isoflavones present in soybean, and it inhibits the Wnt/β-catenin pathway through epigenetic modifications that positively regulate the expression of several Wnt antagonists. It has been demonstrated that treatment with genistein reduced methylation in CpG islands of the Wnt5a and sFRP2 genes, attenuating the nuclear translocation of β-catenin [101,102]. Additionally, the treatment with genistein increased acetylation in the promoter region of the DKK1 gene by histone H3, inhibiting the activity of the Wnt pathway in response to increased expression of the antagonist DKK1 [103]. Finally, mice submitted to the Azoxymethane/Dextran sodium sulfate (AOM/DSS) protocol were used to demonstrate that the functional mechanisms of genistein involve DNA methylation and histone modifications [104]. Genistein also promotes the demethylation of WIF1, another Wnt antagonist, altering the expression of matrix metalloproteases MMP2 and MMP9, molecules essential to invasion and metastasis [105]. Together, these results characterize genistein as an inhibitor of the Wnt/β-catenin signaling with antitumor effect, by inhibiting viability, proliferation and inducing apoptosis and cell cycle arrest in G2.

**Table 1:** Naturally occurring small molecules and their respective molecular targets and mechanisms of inhibition of the Wnt/β-catenin signaling pathway.

Level	Agent	Target	Molecular Mechanism	Reference
Extracellular Domain	Genistein	DKK1 WIF1 sFRP Wnt5a	Upregulation of Wnt antagonists and impairment of the interaction between Wnt-ligand and its receptors	[101,102,103,104,105,106,107]
Destruction Complex	Myricetin Oroxylin A Luteolin Vicenin-2 Jatrorrhizine Raddeanin A	GSK3	Promotes β-catenin phosphorylation and degradation	[108,109]
				[110,111]
				[110,111]
				[112,113]
				[113]
				[114]
	Hydnocarpin	AXIN		[115]
	Berberine	APC		[116,117,118]
	Tussilagone (TSL)	Unknown		[119]
β-catenin	TGG	β-catenin	Promotes β-catenin degradation and impairs its nuclear translocation	[120]
	DIM			[121]
	Curcumin			[122,123,124,125]
	4βHWE			[126]
	EGCG			[127,128]
	Atractylochromene			[129]
	Silibinin			[130,131]
	Broussochalcone			[132]
	Apigenin			[133,134,135]
	Xanthohumol			[136]
	Rabdoternin B			[137]
	Maoecrystal I			
	Wogonin			[138]
	Isoquercitrin			[139]
	Taxifolin			[140,141]
	Derricin			[142]
	Derricidin			
	Antofine			[143]
	Bisleuconothine A			[144]
	Murrayafoline A			[145]
	Tetrandrine			[146]
Transcriptional Machinery	Magnolol Resveratrol Lonchocarpin Piperine Esculetin 2-Hydroxycinnamaldehyde	β-catenin/TCF	Disrupts β-catenin/TCF interaction	[147]
				[148,149,150,151,152]
				[153]
				[154]
				[155]
				[156]
	Carnosic acid	β-catenin/BCL9	Disrupts β-catenin/BCL9 interaction	[157]

### 4.2. Targeting the Destruction Complex

The natural compound tussilagone (TSL) isolated from the flower buds of *Tussilago farfara* is an inhibitor of the Wnt/β-catenin signaling pathway in a range between 10 to 30 µM, downregulating both cytoplasmic and nuclear β-catenin levels in HEK293T reporter cells stimulated by Wnt3a or a GSK3-β inhibitor. TSL promotes proteasome-mediated degradation of the β-catenin since the proteasome inhibitor MG132 recovers the TSL-mediated reduction of the cytosolic level of β-catenin. Additionally, TSL suppressed the proliferation of colon cancer cells via the downregulation of Wnt target genes, such as *CCND1* (cyclin D1) and *MYC* (c-myc) and decreased the cell viability in the MTT assay [119].

The flavonolignan hydnocarpin isolated from *Lonicera japonica* inhibits the Wnt/β-catenin signaling pathway in a dose-dependent manner at concentrations between 5 and 40 µM. Hydnocarpin promotes the upregulation of Axin1/2 and enhances the β-catenin phosphorylation at Ser33/37/Thr41 residues, leading to its subsequent ubiquitin-dependent proteasomal degradation. In addition, it inhibits the proliferation of human colon cancer cells SW480 in the sulforhodamine B (SRB) assay [115]. Raddeanin A is an oleanane-type triterpenoid saponin extracted from the root of *Anemone raddeana* that inhibits the Wnt/β-catenin signaling pathway by the activation of GSK3-β in a concentration less than 5 µM, thereby initiating β-catenin phosphorylation at Ser33/37/Thr41 residues and subsequently, its ubiquitin-dependent proteasomal degradation. Furthermore, raddeanin A reduces cell viability, inhibits cell proliferation, and induces apoptosis in SW480 and LoVo cells in a concentration-dependent manner at concentrations between 0.6 and 1.2 µM. Moreover, raddeanin A efficiently inhibited tumor growth in a xenograft mouse model by reducing the expression of the β-catenin protein and the Wnt-target genes *CCND1* (Cyclin D1) and *MYC* (c-myc) with 2.5 and 5 mg/kg [114].

Myricetin is a flavonoid found in citrus fruits, wild fruits, and grapes [158]. Myricetin inhibits the Wnt/β-catenin pathway by modulating GSK3 activity and reduces cytoplasmic and nuclear β-catenin levels in vivo with 100 mg/kg. In addition, myricetin inhibits the proliferation of intestinal polyps in APC^Min/+^ mice by modulating the localization of β-catenin in intestinal adenomatous cells and negatively regulates the expression of PCNA and the Wnt-target gene cyclin D1, reducing the number of intestinal polyps, besides promoting apoptosis by regulating pro-apoptotic proteins and inflammatory cytokines [108].

The flavone Oroxylin A is present in the tree *Oroxylum indicum* and medicinal plants of the genus *Scutellaria* [109]. Oroxylin A inhibits the activity of the canonical Wnt pathway by downregulating the expression of the Wnt3a protein and promotes the proteasomal degradation of β-catenin in a range between 50 to 150 µM. In addition, this molecule alters the intracellular distribution of β-catenin through the positive regulation of GSK3-β and Axin mediated by intracellular fatty acids. Oroxylin A induces G2/M cell cycle arrest in HCT116 cells, prevents xenographic tumor growth, and reduces the progression of AOM/DSS-induced tumors with 200 mg/kg by modulating fatty acid metabolism. However, the molecular mechanism and the functional effect of this molecule on the Wnt pathway in vivo were not accessed [109].

Luteolin inhibits the canonical Wnt pathway through GKS3 activity, promotes phosphorylation and the subsequent proteasomal degradation of β-catenin dose-dependently in a range between 25 to 100 µM [110,111]. Treatment with different concentrations of luteolin prevents the stabilization of β-catenin, reduces cell viability and proliferation, and induces Caspase-dependent apoptosis in HCT15 cells [110]. Furthermore, luteolin reduces PCNA expression and the Wnt-target gene Cyclin D1 protein level, reducing the proliferation of tumors caused by the AOM/DSS protocol by modulating the localization of β-catenin in mice treated with a single dose with 1.2 mg/kg body weight [111].

Vicenin-2 is the 6,8-di-C-glucoside of apigenin that is widespread in the medicinal plant *Ocimum sanctum*. Yang et al. [112] showed that vicenin-2 dose-dependently inhibits the activity of the reporter gene of the Wnt signaling pathway and represses the inactive form of GSK3, thereby preventing the stabilization of β-catenin in the cytoplasm at 50 µM. Vicenin-2 can also reduce cell viability and proliferation in addition to G2/M arrest and induce Caspase3-mediated cell death in HT-29 cells [112].

Berberine is an isoquinoline alkaloid of medicinal plants of the genus *Coptis* and *Berberis* [118]. Berberine inhibits the activity of the Wnt/β-catenin pathway in a concentration-dependent manner in a range between 5 to 100 µM in cell lines transfected with the TOPFlash reporter plasmid. Berberine does not inhibit the activation promoted by the dnLEFVP16 plasmid. Its likely mechanism of action is by increasing levels of APC, which promotes β-catenin degradation and reduces transcription of target genes such as *CCND1* (cyclin D1) and *MYC* (c-myc) [117,118]. Berberine reduces the viability of cell lines HCT116, KM12L4A, KM12SM, and KM12 and attenuates the formation of intestinal polyps in Apc^Min/+^ mice treated with 330 mg/kg three times per day. Berberine reduced the expression of Wnt-target genes Cyclin D1 and c-Myc in mice, in addition to decreasing the recurrence of polyps in seven patients with familial adenomatous polyposis by reducing Cyclin D1 expression [117,118].

Jatrorrhizine is a protoberberine alkaloid that is one of the main components of *Rhizoma copidis* and has a similar chemical structure to berberine [113,159]. Jatrorrhizine positively regulates GSK3-β expression and promotes a concentration-dependent β-catenin degradation in HCT116 and HT-29 cells in a range between 5 to 15 µM. Jatrorrhizine reduces the viability, proliferation, cell migration and invasion, in addition to inducing S-phase cell cycle arrest and Caspase-dependent apoptosis. Jatrorrhizine also prevents tumor growth and lung metastasis in xenotransplanted mice treated with 2.5 or 5 mg/kg by reducing the β-catenin protein levels [113].

### 4.3. Targeting β-Catenin

1,4,6-Tri-O-galloyl-β-d-glucopyranose (TGG) is a polyphenol isolated from *Sanguisorba officinalis*, a popular Chinese herb called DiYu. TGG significantly inhibits the Wnt/β-catenin signaling pathway by downregulating β-catenin protein levels in a concentration range between 20 to 70 µg/mL. Moreover, TGG induces apoptosis by increasing the protein levels of Caspase-3, PARP and the Bax/Bcl-2 ratio in HT-29 cells [120]. Another natural compound that targets the β-catenin is 3,3′-diindolylmethane (DIM), isolated from cruciferous vegetables. DIM inhibits the Wnt/β-catenin signaling pathway by the downregulation of mRNA and protein levels of c-Myc, β-catenin, and Cyclin D1 in DLD-1 and HCT116 cells in range between 50 to 100 µM. Moreover, DIM inhibits cell growth and clonogenicity, in addition to inducing apoptosis by cleaved-PARP and pro-Caspase-3 of DLD-1 and HCT116 cells [121].

Curcumin, a curcuminoid produced by *Curcuma longa*, inhibits the Wnt/β-catenin signaling pathway in tumor cell lines and mouse models in a dose-dependent manner. Curcumin treatment reduces β-catenin nuclear translocation and decreases transcription of target genes such as *CCND1* (cyclin D1), *MYC* (c-myc), and *AXIN2* (Axin2) in a range between 4 to 32 µM [122,125]. Curcumin also prevents the epithelial-mesenchymal transition, positively regulating NKD2 expression [123]. Furthermore, curcumin induces apoptosis and inhibits tumor cell proliferation and tumor number induced by AOM/DSS protocol by downregulation of the β-catenin protein levels in mice treated orally with 500 mg/kg [122,124,160]. *AXIN2* (Axin2), a known Wnt target gene, was also downregulated in the same mice.

4Hydroxywithanolide E (4βHWE) is a naturally occurring small molecule from *Physalis peruviana* that inhibits the Wnt/β-catenin signaling pathway by promoting the phosphorylation and degradation of β-catenin in a concentration less than 5 µM, which impairs its subsequent nuclear translocation and attenuates expression of the endogenous Wnt target genes in colon cancer cells. 4βHWE reduces cell viability and proliferation, induces G0/G1 cell cycle arrest, and promotes Caspase-dependent apoptosis of colon cancer cells. Moreover, 4βHWE dramatically inhibited tumor growth in HCT116 xenografts by promoting β-catenin degradation and downregulating protein levels of Axin2, c-Myc, and Cyclin D1 in mice treated with 5 mg/kg and 10 mg/kg/day for 14 days, suggesting its potential use as an antitumor agent that targets the Wnt/β-catenin signaling pathway [126].

The flavonoid epigallocatechin-3-gallate (EGCG) is the main component of green tea and reduces the activity of the Wnt/β-catenin pathway induced by the Wnt3a conditioned medium in a range between 20 to 80 µM and promotes phosphorylation of the Ser33/37 sites of β-catenin by a GSK3-independent mechanism [128]. In addition, epigallocatechin-3-gallate can inhibit cell proliferation and the formation of oncospheres enriched with tumor stem cells at 40 µM by downregulating the levels of Axin2, c-Myc, and Cyclin D1 and PCNA [127,128].

Atractylochromene is a polyphenol isolated from rhizomes of *Atractylodes macrocephala* that suppresses the Wnt/β-catenin transcriptional activity stimulated by Wnt3a or GSK3-β inhibitors in HEK293T reporter cells treated with concentrations between 5 to 20 µg/mL. Thus, atractylochromene targets the β-catenin, impairing its galectin-3-mediated nuclear accumulation and inhibiting the proliferation of SW480 colon cancer cells [129].

Silibinin is the active compound present in silymarin extracted from the medicinal plant *Silybum marianum*. Silibinin supplementation at 50 mg/kg/day for 32 weeks reduces β-catenin levels and prevents the colorectal carcinogenesis induced in Wistar rats challenged with the xenobiotic 1,2-Dimethylhydrazine (DMH). Furthermore, silibinin inhibits adenocarcinoma proliferation by downregulating protein levels of β-catenin protein, Cyclin D1 and PCNA [130,131].

Broussochalcone A (BCA) is a prenylated chalcone isolated from *Broussonetia papyrifera* that accelerates the intracellular turnover of β-catenin, leading to its N-terminal phosphorylation at Ser33/37/Thr41 residues and subsequent ubiquitin-dependent proteasomal degradation in a range between 5 to 20 µM. BCA decreased the intracellular β-catenin levels in colorectal cancer cells HCT116 (β-catenin mutant) and SW480 (APC mutant), and the pharmacological inhibition of GSK3-β could not abrogate the BCA-mediated degradation of β-catenin, which suggests that its mechanism of action is downstream of the destruction complex. Moreover, BCA inhibits cell viability and induces apoptosis by a Caspase-dependent mechanism [132].

Apigenin is an abundant flavone in fruits and vegetables that inhibits the reporter activity of the Wnt/β-catenin pathway downstream of the destruction complex in concentrations between 10 to 80 µM [161]. Apigenin changes the distribution of β-catenin and promotes its degradation by the lysosome-dependent autophagy system in a dose-dependent manner [133,134]. In addition, apigenin negatively regulates the expression of several oncogenes and suppresses the proliferation, migration, and invasion of the tumor cell lines SW480 and HCT15 and tumor spheroids at 40 µM [134].

Xanthohumol (XHA) is a natural polyphenol isolated from *Humulus lupulus* that promotes the β-catenin degradation and inhibits cellular proliferation by inhibiting the Wnt/β-catenin signaling pathway activity in mice orally treated with 5 mg/kg of XHA for 8 weeks. Moreover, the administration of XHA suppresses tumor proliferation by downregulating the levels c-Myc, and Cyclin D1 and Ki-67 besides inducing Caspase-3-mediated apoptosis in AOM-induced Sprague-Dawley rats [136].

Rabdoternin B and maoecrystal I, are two natural compounds from a natural ent-kauranoid library identified by a dual-luciferase reporter gene assay. Both compounds inhibit the Wnt/β-catenin signaling pathway in a concentration-dependent manner in HEK293T cells with an IC50 of 13.95 ± 0.66 and 10.58 ± 0.82 µM, respectively. Nevertheless, maoecrystal I did not affect β-catenin levels in SW480 cells, indicating that it inhibits Wnt signaling downstream of β -catenin degradation events. Rabdoternin B and maoecrystal I reduced the viability and inhibited the growth of SW480, HCT116, and HT-29 tumor cells treated with 20 or 40 µM, with only weak cytotoxicity towards the normal colonic epithelial cell line CCD-841-CoN, and arrested SW480 cells at the G2/M phase [137].

Wogonin is a flavone present in medicinal plants of the *Scutellaria* genus that inhibits the Wnt/β-catenin pathway in a dose-dependent manner in HCT116 cells treated with concentrations between 10 to 40 µM. Wogonin promotes the reduction of cytoplasmic and nuclear β-catenin levels and downregulates the expression of transcription factors of the TCF/LEF1 family, thereby limiting the transcriptional capacity of the TCF/LEF complex. Wogonin reduces cell viability and proliferation, induces cell cycle arrest in G1, and reduces the growth of xenographic tumors by promoting β-catenin degradation and downregulating protein levels of c-Myc and Cyclin D1 in mice treated 30 and 60 mg/kg [138].

Isoquercitrin, a flavonol derived from quercetin, inhibits the activity of the reporter gene for the Wnt/β-catenin pathway in a concentration-dependent manner, promoting β-catenin degradation, and preventing its translocation to the nucleus in a range between 75 to 150 µM. As a result, isoquercitrin inhibits the formation of the ectopic axis induced by *xWnt8* (Xenopus *Wnt8* mRNA) and the dorsalization phenotype induced by lithium chloride (LiCl) in *Xenopus laevis* embryos. In addition, isoquercitrin reduces the viability, proliferation, and migration of tumor cell lines HCT116, SW480, and DLD-1 without affecting the non-tumor cell line IEC-18 [139].

Taxifolin is a flavonoid abundant in olive oil, onions, grapes, and various citrus fruits. Taxifolin interacts with β-catenin and reduces its total levels, thereby modulating the canonical Wnt signaling in a concentration-dependent manner in concentrations between 20 to 60 µM [140,141]. In addition to stimulating G2 arrest and inducing apoptosis through the Caspase pathway, taxifolin can also reduce cell viability and proliferation in HCT116 and HT-29 cell lines and xenographic tumors by the downregulation of the β-catenin and Cyclin D1 in mice treated with 0.4 µg/kg [140,141].

The chalcones derricin and derricidin inhibit the Wnt/β-catenin signaling pathway with different mechanisms [142]. Both inhibit the activation of the Wnt reporter in a dose-dependent manner. However, derricin inhibits the mutant S33A of β-catenin and probably prevents its nuclear translocation. On the other hand, derricidin does not inhibit this mutant, suggesting that its mechanism of action is upstream of β-catenin. Functional assays showed that derricin and derricidin disrupt the axial pattern and reverse the ectopic axes induced by *xWnt8* DNA in embryos of *X. laevis*, reinforcing their inhibitor properties in a well-established canonical Wnt pathway model [162]. Furthermore, both chalcones have antitumor effects and inhibit viability, proliferation, migration, and induce cell cycle arrest in colorectal tumor cell lines [142].

Antofine is an alkaloid phenanthroindolizidine present in several plants of the Asclepiadaceae family [143]. Antofine reduces the levels of β-catenin and Cyclin D1 in SW480 cells and inhibits the activity of the Wnt/β-catenin pathway in a concentration-dependent manner in HCT116 cells transfected with the TOPFlash plasmid in 2.5 to 10 nanomolar range of concentration. Furthermore, treatment with antofine prevented cell cycle progression in the G0/G1 phases by disrupting the Cyclin/CDK complex. Antofine also induces apoptosis and prevents the growth of xenotransplanted tumors in nude mice. However, neither the mechanism nor the functional effect of antofine on the Wnt/β-catenin pathway in vivo was accessed [143].

Bisleuconothine A is an alkaloid bisindole that promotes β-catenin degradation, preventing its nuclear translocation and reducing the activity of the Wnt/β-catenin signaling pathway. Bisleuconothine A reduces the transcriptional activity of the Wnt canonical pathway in a concentration-dependent manner in HCT116 and SW480 cells and induces β-catenin phosphorylation impairing its nuclear levels after treatments with concentrations between 1 to 10 µM. Furthermore, Bisleuconothine A inhibits cell proliferation by arresting the cell cycle in G0/G1 and inducing Caspase-dependent apoptosis and suppresses the growth of xenotransplanted tumors in mice, promoting the β-catenin degradation and downregulating protein levels of c-Myc and Cyclin D1 in mice treated with 2 mg/kg for 2 weeks [144].

Murrayafoline A is a carbazole alkaloid isolated from *Glycosmis stenocarpa* that inhibits Wnt/β-catenin signaling pathway according to its concentration. Murrayafoline A inhibits the pathway activated by Wnt3a conditioned medium or LiCl treatment in HEK293T cells treated with concentrations between 5 to 40 µM and promotes β-catenin degradation by a proteasome-dependent mechanism. Furthermore, murrayafoline A down-regulates the expression of c-Myc and Cyclin D1 and reduces the viability and proliferation of human colon tumor lines [145].

Tetrandrine, a bisbenzylisoquinoline alkaloid extracted from *Stephania tetrandra* roots, inhibits the Wnt/β-catenin pathway reporter gene activity in LoVo and HCT116 cells treated with concentrations between 2.5 and 10 µM [163,164]. Tetrandrine reduces the expression of *MYC* (c-myc), a target gene of the Wnt canonical pathway, inhibits cell proliferation and migration, in addition to inducing Caspase-dependent apoptosis. Tetrandrine also prevents tumor growth of xenotransplanted tumors in rats treated intraperitoneally with 60 mg/kg once every 2 days [163] and DMH-DSS induced tumors in mice orally treated with 40 or 80 mg/kg [164].

### 4.4. Targeting the β-Catenin Transcriptional Activity

Magnolol is a neolignan from the cortex of *Magnolia obovata* that suppresses the Wnt/β-catenin transcriptional activity stimulated by Wnt3a in HEK293T reporter cells. Mechanistically, magnolol inhibits the nuclear translocation of β-catenin and significantly suppresses the binding of β-catenin/TCF complexes onto their specific DNA-binding sites in the nucleus. Magnolol also effectively inhibits the proliferation of both SW480 and HCT116 cells in a concentration-dependent manner and suppresses the growth and invasion of HCT116 xenografts in a nude mouse model [147].

Resveratrol is a flavonoid with several biological properties. It is present in peanuts and wild fruits and is abundant in grapes and their derivatives, such as red wine. Resveratrol inhibits the Wnt/β-catenin pathway in a dose-dependent manner, preventing the interaction between β-catenin/TCF4 and promoting proteasomal degradation of TCF4 in a range between 10 to 100 µM [148,151]. Resveratrol also inhibits the proliferation, migration, and invasion of tumor cell lines and xenotransplanted tumors in response to down-regulation of MALAT1 [150], HMGA2 [149], and the PTEN/PI3K/Akt axis in mouse treated with 2.5 and 25mg/kg of resveratrol [152].

Lonchocarpin is a chalcone that inhibits the Wnt/β-catenin pathway with an IC50 of 4 µM. Lonchocarpin reduces the nuclear level of β-catenin and inhibits dnTCF4 VP16-induced activation in HEK293T cells, suggesting that its mechanism of action involves disturbing the interaction between β-catenin and TCF/LEF transcription factors. In functional assays, lonchocarpin disturbed the axial patterning and reversed the Wnt8-induced ectopic axes in *X. laevis* embryos. Furthermore, lonchocarpin inhibits viability, proliferation, and migration in human colorectal tumor lines, in addition to reducing tumor proliferation in mice submitted to the AOM/DSS protocol [153].

Piperine is an alkaloid responsible for the spicy taste of black pepper (*Piper nigrum*) and long pepper (*Piper longum*) that inhibits the transcriptional machinery of the Wnt/β-catenin pathway at 34 µM in SW480 β-catenin reporter gene cells and reduces the nuclear localization of β-catenin in HCT116 cells. Piperine impairs colorectal cancer progression by decreasing cell viability, proliferation, and migration in human tumor lines HCT116, SW480, and DLD-1. Still, its effects are attenuated both in RKO cells, a colorectal carcinoma lineage that lacks an overactive Wnt pathway, and HEK293T *CTNNB1* (gene that encodes β-catenin) knockout cells, reinforcing its role as a selective inhibitor of the canonical Wnt pathway [154].

Esculetin (6,7-dihydroxycoumarin) is a natural small-molecule derivative of coumarin identified as a potential inhibitor of the Wnt/β-catenin signaling pathway in concentrations between 20 to 80 µM. The interaction between esculetin and β-catenin inhibits the formation of the β-catenin-TCF complex, suppressing the Wnt/β-catenin transcriptional activity. Moreover, esculetin effectively decreased the cell viability and inhibited tumor growth of HCT115, HCT116, and DLD-1 cells, and impaired tumor growth in a colon cancer xenograft mouse model by reducing protein levels of Cyclin D1, c-Myc and Ki-67 [155].

The carnosic acid from rosemary attenuates the transcriptional activity of the Wnt/β-catenin pathway in colorectal cancer cells with concentrations between 2.5 to 25 µM. Nuclear magnetic resonance spectroscopy and analytical ultracentrifugation show that the carnosic acid response requires an amino-terminal intrinsically labile α-helix (H1) next to the BCL9-binding site in β-catenin. Carnosic acid targets predominantly the oncogenic form of β-catenin for proteasomal degradation in an H1-dependent manner, providing a new strategy for developing target inhibitors of oncogenic β-catenin [157].

The phenylpropanoid compound 2-Hydroxycinnamaldehyde (phenylpropanoid 1) isolated from the bark of *Cinnamomum cassia,* significantly inhibited the Wnt/β-catenin signaling pathway in HEK293T cells and HCT116 colorectal cancer cells. Mechanistically, phenylpropanoid 1 suppresses the β-catenin/TCF binding complex into the nucleus and down-regulates Wnt target genes such as *MYC* (c-myc) and *CCND1* (cyclin D1). Furthermore, the compound inhibits tumor cells proliferation in the athymic xenograft mice model, without any apparent toxicity yet suppressing the expression of Wnt target genes associated with tumor growth, including *MYC* (c-myc), *CCND1* (cyclin D1), and *BIRC5* (survivin) [156].

## 5. Concluding Remarks and Future Perspectives

The Wnt canonical signaling pathway plays a prime role in regulating the levels of the β-catenin protein and is vital for the embryonic axes patterning and maintaining stem cells from different tissues [29,31]. It is therefore foreseeable that changes in its components lead to a pathological process such as CRC. According to the TCGA, more than 90% of CRC cases present dysregulation in regulatory proteins, such as the loss of the tumor suppressor APC [65]. As a result, there is constitutive activation of the pathway, which leads to an abnormal proliferative event and to the progression of CRC [63,73,79].

CRC ranks second in the number of cancer deaths worldwide, mainly due to late diagnoses, restricting treatment in the potentially curable stages, and decreasing patient survival [2,4,11,12,13]. The treatment of CRC involves surgery to remove the tumor tissue, in addition to radiotherapy and systemic chemotherapy sessions based on the use of 5-FU [83,84,85,86]. However, almost half of patients are resistant to these treatments, especially in metastatic cases [89], where the 5-year survival rate is only 12% [14,15]. This factor may be related to the intratumoral heterogeneity, TME, and the presence of CSCs, which is impossible to resolve with the standard approaches currently available in clinical practice.

Myofibroblasts, cancer-associated fibroblasts (CAFs), and CSCs secrete a range of growth factors and signaling proteins such as the agonist Wnt3a and the antagonist Notum in the TME [24,165], both essential for canonical Wnt signaling transduction. Recently, a new mechanism for tumor resistance was proposed, suggesting that APC-deficient cell clones become “supercompetitive” through TME modulation, secreting Wnt antagonists such as Notum, which inhibit the survival of normal ISC neighboring cells, inducing their differentiation [79,80,81]. Thus, the use of selective Notum inhibitors, such as the small molecule LP922056 [166], becomes a hypothetical route to prevent the fixation and expansion of APC-mutant clones in patients predisposed to the development of CRC.

The proliferative-related genes *MYC* (c-myc)***,*** *AXIN2* (Axin2)*,* and *CCND1* (cyclin D1) are commonly linked to CRC development and are considered targets of the canonical Wnt pathway, in which APC is a prime regulator [167]. Most APC mutations in CRC are nonsense mutations, which result in frameshift or the introduction of a premature stop codon [68,70]. Therefore, the use of APC-directed strategies could represent a promising route for CRC therapies. According to Zilberberg and colleagues, restoration of even 1% APC function in colorectal cancer cells by aminoglycoside- and macrolide-induced read-through of premature termination codons may result in near-normal or a clinically less severe CRC phenotype [168]. Moreover, Dow and colleagues showed that colon cancer cells recovered their normal function after restoring APC levels of APC-truncated cancer cells harboring KRAS and p53 mutations [169].

Despite promising advances in the use of Wnt/β-catenin signaling modulators in experimental therapies against CRC, no molecules of natural or synthetic origin are clinically available [170]. The principal obstacle is the difficulty of inhibiting only the aberrant activation, as the Wnt signaling pathway is essential for cellular homeostasis throughout the body [19,20,60,171]. Currently, the only Wnt inhibitor in clinical testing is the LGK974 molecule (NCT01351103), a Porcupine inhibitor that prevents the secretion of the Wnt3a ligand [172]. Although promising, this molecule may have limitations in clinical use for CRC as most cases bear APC mutations, and the downstream signaling responsiveness of the Wnt ligand in CRCs harboring the loss of function of the tumor suppressor APC remains unknown [25].

Combination therapy is a good strategy to overcome tumor resistance, and Wnt/β-catenin inhibitors have been shown to improve tumor sensitivity to 5-FU and oxaliplatin, which are standard drugs in regimens such as FOLFOX, used by CRC patients in stages III and IV [23,25,173]. Low curcumin doses enhance 5-FU effects against colorectal cancer cells and dramatically reduce the number and volume of tumors in mice subjected to the colitis-associated CRC protocol [174,175,176,177]. Combined 5-FU with thymoquinone in sublethal doses was effective against CD133 + tumor stem cells and showed no toxic effects on healthy organoids [178]. Additionally, many other naturally occurring small molecules used as Wnt inhibitors have had promising effects when combined with chemotherapeutic agents, the most observed effects being inhibition of proliferation and induction of apoptosis [179,180,181,182,183,184,185,186]. Therefore, combining Wnt inhibitor drugs with current chemotherapeutic treatment might be an interesting approach to overcome CRC carcinogenesis.

In this review, we evaluated the use of small molecules of natural origin to inhibit the Wnt/β-catenin signaling pathway in experimental models of CRC and identified several molecules with high inhibitory potential. However, some papers had inconclusive information from the mechanistic perspective, which might be related to the incompatible or improper experimental design to address mechanistic questions. It is, therefore, necessary to overcome these issues with more suitable experimental models that enable a reliable characterization of their mechanisms of action and potential application in further preclinic studies.

So far, we have reviewed dozens of promising naturally occurring small molecules that differently inhibit the canonical Wnt signaling pathway. Considering the mechanisms of action of each compound and the dysregulation of Wnt signaling in the different colorectal cancer types, novel pre-clinical experiments should be performed to validate and perhaps allow these small molecules to move off the laboratory bench onto the clinical platform.
